# Acute myocardial infarction activates distinct inflammation and proliferation pathways in circulating monocytes, prior to recruitment, and identified through conserved transcriptional responses in mice and humans

**DOI:** 10.1093/eurheartj/ehv195

**Published:** 2015-05-16

**Authors:** Neil Ruparelia, Jernej Godec, Regent Lee, Joshua T. Chai, Erica Dall'Armellina, Debra McAndrew, Janet E. Digby, J. Colin Forfar, Bernard D. Prendergast, Rajesh K. Kharbanda, Adrian P. Banning, Stefan Neubauer, Craig A. Lygate, Keith M. Channon, Nicholas W. Haining, Robin P. Choudhury

**Affiliations:** 1 Division of Cardiovascular Medicine, Radcliffe Department of Medicine, John Radcliffe Hospital, University of Oxford, Headley Way, Oxford OX3 9DU, UK; 2 Oxford Heart Centre, John Radcliffe Hospital, Headley Way, Oxford OX3 9DU, UK; 3 Dana-Farber Cancer Institute, Harvard Medical School, 44 Binney Street, Boston, MA 02115, USA; 4 Acute Vascular Imaging Centre, Radcliffe Department of Medicine, John Radcliffe Hospital, University of Oxford, Headley Way, Oxford OX3 9DU, UK

**Keywords:** Acute myocardial infarction, Monocytes, Genomics, Inflammation, Mitosis

## Abstract

**Aims:**

Monocytes play critical roles in tissue injury and repair following acute myocardial infarction (AMI). Specifically targeting inflammatory monocytes in experimental models leads to reduced infarct size and improved healing. However, data from humans are sparse, and it remains unclear whether monocytes play an equally important role in humans. The aim of this study was to investigate whether the monocyte response following AMI is conserved between humans and mice and interrogate patterns of gene expression to identify regulated functions.

**Methods and results:**

Thirty patients (AMI) and 24 control patients (stable coronary atherosclerosis) were enrolled. Female C57BL/6J mice (*n* = 6/group) underwent AMI by surgical coronary ligation. Myocardial injury was quantified by magnetic resonance imaging (human) and echocardiography (mice). Peripheral monocytes were isolated at presentation and at 48 h. RNA from separated monocytes was hybridized to Illumina beadchips. Acute myocardial infarction resulted in a significant peripheral monocytosis in both species that positively correlated with the extent of myocardial injury. Analysis of the monocyte transcriptome following AMI demonstrated significant conservation and identified inflammation and mitosis as central processes to this response. These findings were validated in both species.

**Conclusions:**

Our findings show that the monocyte transcriptome is conserved between mice and humans following AMI. Patterns of gene expression associated with inflammation and proliferation appear to be switched on *prior* to their infiltration of injured myocardium suggesting that the specific targeting of inflammatory and proliferative processes in these immune cells in humans are possible therapeutic strategies. Importantly, they could be effective in the hours *after* AMI.

## Abstract (precis)

The immune system plays critical roles in myocardial injury and repair following acute myocardial infarction (AMI). Evidence from experimental models strongly implicates monocytes as critical to these processes and their specific targeting results in a significant reduction in infarct size and improved healing. It is currently unclear if monocytes play a similarly important role in humans. Examining changes in the patterns of gene expression can address this question. Here, we show that the peripheral blood monocyte response following AMI is conserved between species and that monocytes appear to be ‘programmed’ prior to their arrival at sites of myocardial injury. This investigation may translate to the future development of therapeutics to treat patients presenting with AMI that importantly would be effective in the hours *after* the onset of ischaemia.

**Translational perspective**

The immune system plays critical roles in myocardial injury and repair following acute myocardial infarction (AMI). Evidence from experimental models strongly implicates monocytes as critical to these processes and their specific targeting results in a significant reduction in infarct size and improved healing. It is currently unclear if monocytes play a similarly important role in humans. Examining changes in the patterns of gene expression can address this question. Here, we show that the peripheral blood monocyte response following AMI is conserved between species and that monocytes appear to be ‘programmed’ prior to their arrival at sites of myocardial injury. This investigation may translate to the future development of therapeutics to treat patients presenting with AMI that importantly would be effective in the hours *after* the onset of ischaemia.

## Introduction

In AMI, irreversible tissue injury occurs due to sustained ischaemia. In addition, recent pivotal studies have shown that the innate immune system is activated in AMI, sequentially mediating aspects of both injury and repair.^1–3^ Importantly, early phase monocytes appear to act as *mediators* of injury, since attenuating the response of inflammatory monocytes with siRNA,^4,5^ angiotensin converting enzyme inhibitors,^6^ or splenectomy^6^ significantly reduced infarct size in experimental models.^4–6^ These processes have been studied in great detail in mice^5–8^ but analogous data from humans are sparse^9,10^ and it is therefore not clear if monocytes in humans are equally important in comparison with experimental models.

In fact, for both humans and mice, characterization of the monocyte response following AMI has largely relied on the analysis of a very limited repertoire of cell surface proteins.^11,12^ In mice, monocytes are functionally and phenotypically heterogeneous and can be divided into subsets by the presence of the cell surface protein Ly6C. Ly6C^hi^ monocytes express high levels of CCR2 and accumulate preferentially in inflammatory sites.^11^ These can be distinguished from Ly6C^lo^ monocytes, which patrol the vasculature^13^ and have roles in tissue repair and angiogenesis.^7^ Following AMI in mice, and possibly humans,^10,14^ monocytes from the spleen^15^ and the bone marrow^16^ are recruited to ischaemic myocardium via the blood in a coordinated manner.^9^ In mice, Ly6C^hi^ monocytes are mobilized early, peaking in blood at ∼48 h. There is also monocyte heterogeneity in humans, with analogous subsets based on the cell surface protein expression of CD14 and CD16.^12^ CD14^++^CD16^−^ resemble Ly6C^hi^ monocytes, whilst CD14^dim^CD16^+^ monocytes resemble Ly6C^lo^ monocytes in mice.

Whilst cell surface proteins distinguish monocyte subsets in mice and humans, are of descriptive value and are widely used, they provide little functional insight. In contrast, whole-genome expression profiling in leukocytes provides an opportunity for unbiased analysis of cellular function and therefore of the characterization of pathways and processes with far greater complexity than can be attained using only surface markers.^17–19^ Accordingly, we quantify changes in the transcriptome of monocytes isolated from peripheral blood early after AMI, as well as in absolute monocyte numbers (and their subsets).

Mouse models can be highly informative and have enabled increased understanding in the pathogenesis of AMI. However, cautionary experience from other models argues that they cannot be assumed to be representative of human disease.^20,21^ By comparing changes in the monocyte transcriptome following AMI in mice and humans, we evaluate whether there is a conserved response between species, thereby contributing to the validation of mouse models for the study of disease mechanisms and therapies in this domain. Furthermore, we use gene set enrichment analysis (GSEA) to determine and understand the functional relevance of these responses, which we confirm at protein level, thereby identifying new possibilities for patient stratification and targeted therapies post AMI.

## Materials and methods

### Ethics statement

Animal studies were undertaken with the approval of the University of Oxford Ethical Review Committee and procedures were conducted in accordance with the UK Home Office Animals (Science Procedures) Act 1986 (HMSO UK) incorporating European directive 2010/63/EU. The clinical study protocols were approved by the Oxfordshire Research Ethics Committee (references 08/H0603/41 and 11/SC/0397). All patients provided informed consent to participate.

### Mouse model of acute myocardial infarction

All experiments were conducted on female C57BL/6J mice (Harlan, Blackthorn, UK) at 24 weeks of age (*n* = 6/group) due to the higher incidence of acute ventricular rupture in male mice.^22^ Surgical AMI was induced as previously described.^23^ Sham-procedure mice underwent the same protocol, but without ligation of the coronary artery. Transthoracic echocardiography (TTE) was carried out with a Vevo 2100 ultrasound system (Visualsonics, Amsterdam, The Netherlands) 48 h after AMI and fractional shortening was used to quantify infarct size of infarct.

### Human subjects

Thirty consecutive patients presenting to the Oxford Heart Centre with STEMI were recruited between June 2012 and November 2012 as part of the Oxford acute myocardial infarction (OxAMI) study. Peripheral venous blood was obtained from patients at presentation (the ‘hyperacute’ time point—*before* primary angioplasty and during AMI) and at 48 h following presentation (48 h time point) and processed within 30 min. Additionally, 24 stable patients, with confirmed coronary atherosclerosis were recruited to act as controls (*Table 1*).

**Table 1:** Patient demographic data

	CONTROL	STEMI	*P*-value
*n*	24	30	n/a
M:F	18:6	27:3	*P* = 0.71
Age (mean, range)	63.4 (46–79)	60.1 (38–87)	*P* = 0.64
CV risk			
Diabetes mellitus	3 (12.5%)	4 (13.3%)	*P* = 0.81
Smoker	13 (54.2%)	19 (63.3%)	*P* = 0.42
Hypertension	21 (87.5%)	10 (33.3%)	*P* < 0.001
Hypercholesterolaemia	10 (41.7%)	4 (13.3%)	*P* < 0.001
Family history	10 (41.7%)	12 (40%)	*P* = 0.53
Previous stroke/AMI	11 (45.8%)	0 (0%)	*P* < 0.001
Lipoproteins (mmol/L) (mean, SD)			
Total cholesterol	4.14 (1.09)	4.74 (0.87)	*P* = 0.77
LDL-c	2.2 (0.82)	3.1 (1.23)	*P* = 0.26
HDL-c	1.09 (0.29)	1.07 (0.3)	*P* = 0.89
TG	1.78 (0.85)	1.5 (1.28)	*P* = 0.19
Admission medication			
Aspirin	n/a	1 (3.3%)	n/a
Clopidogrel/prasugrel	n/a	0 (0%)	n/a
β-Blocker	n/a	2 (6.7%)	n/a
ACE-I/ARB	n/a	5 (16.7%)	n/a
Statin	n/a	4 (13.3%)	n/a
Calcium blocker	n/a	5 (16.7%)	n/a
Diuretic	n/a	1 (3.3%)	n/a
Renal function (mean, SD)			
Creatinine (μmol/L)	85.54 (19)	82.23 (14.95)	*P* = 0.24
Infarct-related artery			
Left main stem	n/a	0 (0%)	n/a
Left anterior descending	n/a	14 (46.7%)	n/a
Circumflex	n/a	4 (13.3%)	n/a
Right coronary artery	n/a	12 (40%)	n/a
Peri-procedural medication			
Bivalirudin	n/a	30 (100%)	n/a
Heparin	n/a	16 (53.3%)	n/a
Glycoprotein IIb/IIIa inhibitor	n/a	0 (0%)	n/a
Revascularization time (min)			
Call-to-balloon	n/a	119.4 (47.93)	n/a
Door-to-balloon	n/a	35.48 (32.25)	n/a
Discharge medication			
Aspirin	24 (100%)	30 (100%)	*P* = 1.0
Clopidogrel/prasugrel	10 (41.7%)	30 (100%)	*P* < 0.001
β-Blocker	17 (70.8%)	29 (96.7%)	*P* < 0.01
ACE-I/ARB	18 (75%)	29 (96.7%)	*P* < 0.01
Statin	17 (70.8%)	30 (100%)	*P* < 0.01
Calcium blocker	7 (29%)	0 (0%)	*P* < 0.001
Diuretic	3 (12.5%)	4 (13.3%)	*P* = 0.93

STEMI, ST-segment elevation myocardial infarction; *n*, number; M, male; F, female; CV, cardiovascular; LDL-c, low-density lipoprotein cholesterol; HDL-c, high-density lipoprotein cholesterol; TG, triglycerides; ACEI, angiotensin converting enzyme inhibitor; ARB, angiotensin receptor blocker.

All patients (*n* = 30) underwent 3 Tesla cardiac magnetic resonance (CMR; Verio, Siemens, Germany) within 48 h of presentation and 23 patients underwent a second CMR scan at 6 months. Both CMR scans assessed left ventricular function (LVF), oedema, and late gadolinium enhancement (LGE) as previously described^24^ (Supplementary material online, *Table S2*). Cardiac magnetic resonance analysis was carried out using cmr42^®^ software (Circle Cardiovascular Imaging Inc., Calgary, Canada).

### Flow cytometry

All stained samples were analysed using the flow cytometer (CyAN ADP Flow cytometer, Dako, Ely, UK). Data were analysed using FlowJo software, version 7.6.3 (Tree Star Inc., OR, USA).

### Mice

Whole blood was obtained (*n* = 6/group) and cell suspensions were prepared as previously described^25^ and were incubated with the following antibodies: CD11b-APC, Ly6G-PE, Ly6C-FITC (all BD Pharmingen, Oxford, UK), and CD115-PerCP (eBioscience, San Diego, USA).

### Human

Whole venous blood was stained with the following antibodies: CD14-APC, CD16-PE-Cy7, CD86-PE, CD42b-FITC, CD11b-Pacific Blue, CD66b-PE-Cy5, CD56-PE-Cy5, CD123-PE-Cy5 (all BD Pharmingen), and TLR2-FITC (all BD Pharmingen) or permeabilized and stained with Ki67-FITC (BD Pharmingen) for validation studies.

### Monocyte cell sorting

Mouse cell suspensions were immediately sorted on an MOFLO cell sorter (Dako, Ely, UK). Human monocytes were isolated using the EasySep Human monocyte enrichment kit without CD16 depletion (StemCell Technologies, Grenoble, France) as per the manufacturer's instructions.

### Microarray

Extracted and purified RNA samples from individual samples with a 260/280 > 2.0 and an RNA integrity number >7.0 (Agilent, Wokingham, UK) were deemed acceptable. Following amplification and biotin labelled, mouse RNA was hybridized to Mouse WG-6 BeadChips (Illumina, San Diego, CA, USA) and human RNA to Illumina Human HT12v3.0 BeadChips (Illumina).

### Immunohistochemistry

Frozen sections of tissue harvested from mice were stained with CD11b (BD Pharmingen, Oxford, UK) and Ki67 (Abcam, Cambridge, UK). Five random FOVs at 20× magnification were recorded (Leica DM2500 microscope) and the number of cells positive for CD11b and Ki67 were assessed.

### Analysis of gene expression profiles

Microarray data were analysed using GenePattern (Broad Institute, Cambridge, MA, USA). Differentially expressed genes were identified using an unpaired *t*-test with a fold change of >1.5 and a *P* < 0.05 deemed necessary for significance. Gene-set enrichment analysis and leading-edge analysis using the C7 collection of the molecular signature database (MSIGDB; http://www.broadinstitute.org/gsea/msigdb) was performed as previously described,^26,27^ a false discovery rate (FDR) < 0.25 was deemed necessary for significance.

### Gene set enrichment analysis

Gene set enrichment analysis yields a quantitative measure of the over-representation of a set of genes (e.g. genes encoding products in a same metabolic pathway) at the top or bottom of a ranked list of genes. Candidate genes are ranked by their differential expression between two phenotypes. The statistic is a weighted Kolmogorov–Smirnov-like statistic and significance is calculated using an empirical permutation test.^28^ Here we applied an extended version of conventional GSEA in order to produce an enrichment score in a single sample, as described previously.^29^ Such a score is necessary in order to make a predictive call on single samples without reference to a larger group of samples. In this approach, the genes are ordered based on either absolute expression or the relative changes with respect to the baseline level.

Gene set enrichment analysis was performed as described previously.^28^ Gene sets enriching with FDR < 0.01 were used for leading-edge analysis using Pearson correlation.^30^ Gene clusters were functionally annotated by calculating overlap with GO terms using PANTHER algorithm^31^ on AmiGO 2 version 2.1.3.^32,33^

### Statistics

Values are expressed as mean ± SD. The Gaussian distribution of all parameters was tested. Differences in continuous variables between groups were compared using the Student *t*-test. Categorical variables are presented as numeric values and percentages. Correlative analyses were determined by Spearman statistical test to determine the percentage of variance explained or a spline fit for non-linear relationships. All statistical tests were two-tailed and a *P* < 0.05 was considered statistically significant. Analyses were performed with GraphPad Prism Version 5 (GraphPad Software Inc., San Diego, CA, USA) and SPSS Version 21 (IBM Corporation, NY, USA).

## Results

### Acute myocardial infarction results in a peripheral monocytosis at 48 h

**Figure 1:**
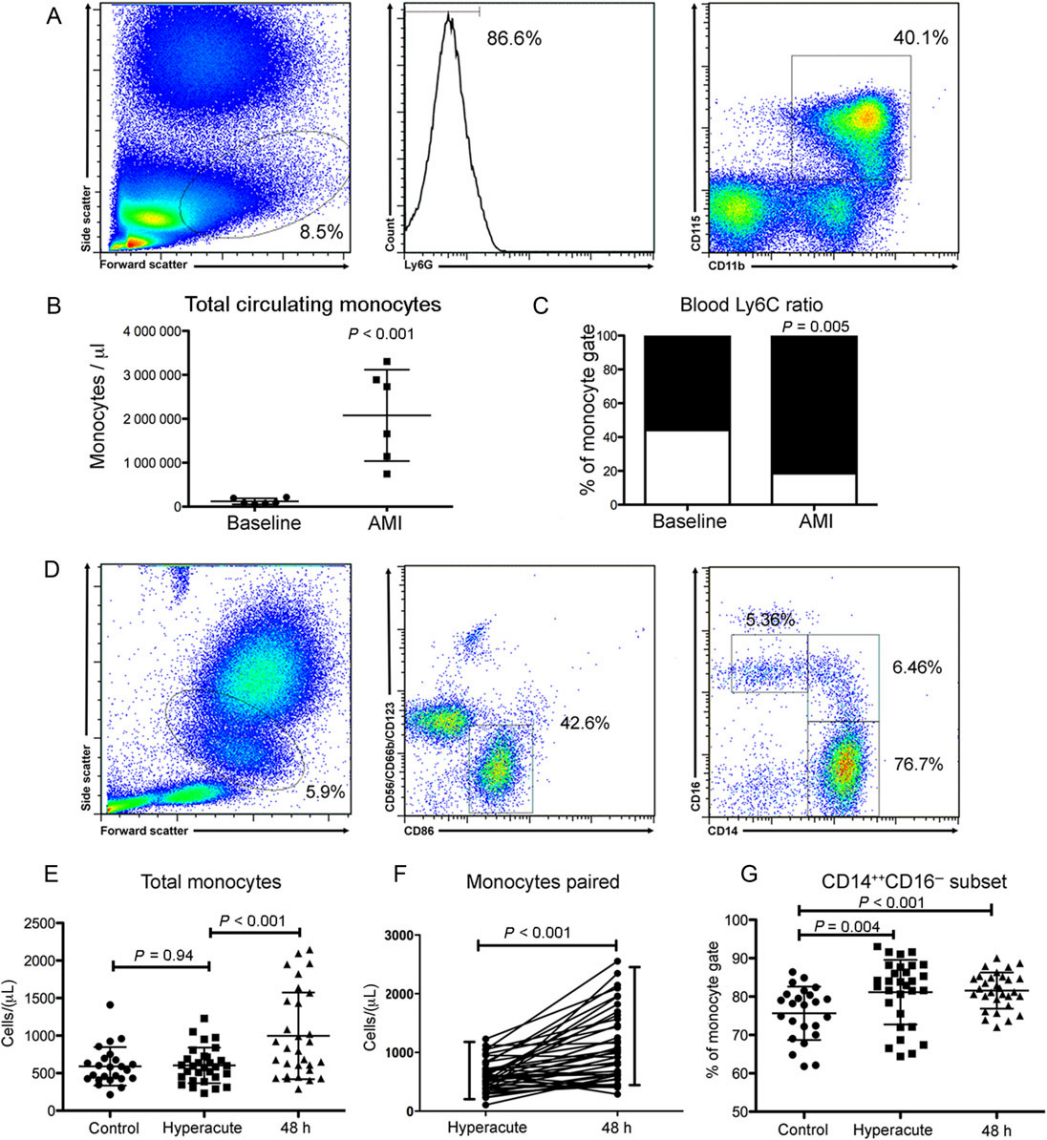
Acute myocardial infarction results in an increase in total peripheral circulating monocytes of an inflammatory phenotype in both mice and humans. (*A*) Fluorescence-activate cell sorting (FACS) gating strategy to identify monocytes in peripheral blood (mice, *n* = 6/group). (*B*) Acute myocardial infarction results in a significant 4-fold increase in total monocytes (*P* < 0.001), which were predominately of an inflammatory Ly6C^hi^ phenotype (*C*). (*D*) Human monocyte (and monocyte subset) gating strategy (*n* = 24 control group, *n* = 30 acute myocardial infarction group). (*E*) Acute myocardial infarction results in a significant 2-fold increase in total monocytes (*P* < 0.001) at 48 h following injury but not at the hyperacute (at presentation) timepoint (*F*). (*G*) Monocytes at both the hyperacute and 48 h time points exhibited an inflammatory CD14^++^CD16^−^ phenotype. Data are represented by mean ± standard deviation.

Following AMI in the mouse there was an increase (4-fold; *P* < 0.001) in total peripheral circulating monocytes at 48 h (*Figure 1A* and *B*). Similarly, in humans, flow cytometry of whole blood 48 h following onset of symptoms showed a 2-fold increase (*P* < 0.001) in total circulating monocytes in comparisons with both (i) the hyperacute time point and (ii) stable control subjects (*Figure 1D*–*F*). There was no significant difference in total peripheral circulating monocytes at presentation (hyperacute time point) compared with stable controls (*Figure 1E*).

### Monocytes display an inflammatory phenotype 48 h following acute myocardial infarction

In the mouse model of AMI at 48 h, monocytes were predominantly of the Ly6C^hi^, inflammatory subset (*P* = 0.005, *Figure 1C*). Similarly, in human patients there was a small, but statistically significant, CD14^++^CD16^−^ inflammatory subset preponderance at 48 h (82.9 ± 6.9% vs. 75.9 ± 5.1% (*P* < 0.001), compared with control patients with stable atherosclerosis (*Figure 1G*).

### The magnitude of the monocyte response in both mice and humans correlates with the extent of myocardial injury

**Figure 2:**
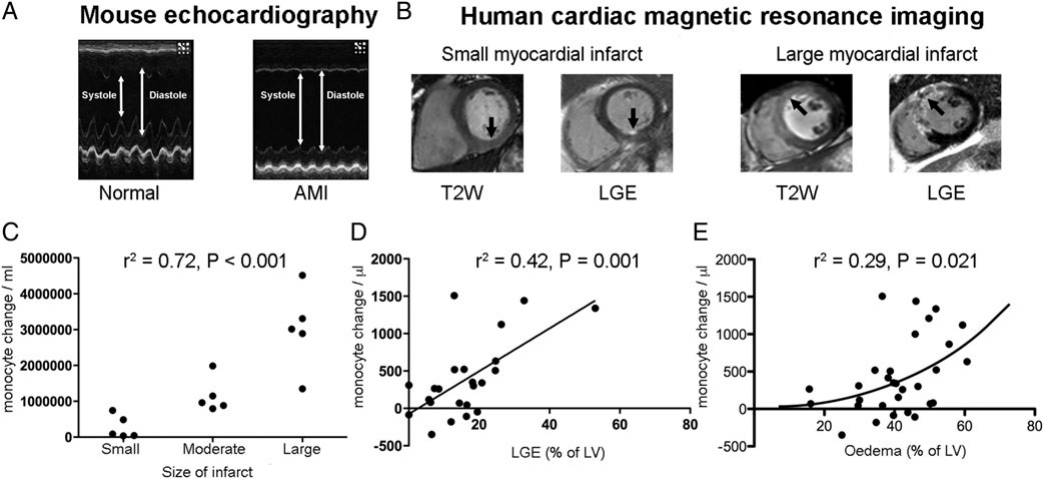
The extent of the monocyte response correlates with the extent of myocardial injury. (*A*) Transthoracic echocardiography of mouse hearts following acute myocardial infarction (*n* = 6/group). (*B*) Cardiac magnetic resonance imaging to quantify area of injury (T2-weighted sequence) and infarction (LGE sequence) in humans (*n* = 30 acute timepoint, *n* = 23 follow up). (*C*) In mice, the total peripheral monocyte count was highly correlated with size of infarct (*r*^2^ = 0.72, *P* < 0.001). (*D*) In humans, the magnitude of the monocyte response for each patient following acute myocardial infarction correlated with the extent of irreversible myocardial injury determined by LGE at 6 months (*r*^2^ = 0.42, *P* = 0.001) and weakly (non-linear relationship) with the area of risk, as measured by oedema (*r*^2^ = 0.29, *P* = 0.021, *E*).

Due to the anticipated marked variation in infarct size in mice that underwent AMI, mice were analysed by tertiles of infarct size, as characterized by TTE (*Figure 2A*). The total peripheral monocyte count was highly correlated with size of infarct (*r*^2^ = 0.72, *P* < 0.001, *Figure 2C*). Similarly, in humans, the magnitude of the monocyte response for each patient (the difference between the 48 h monocyte count and the hyperacute monocyte count) following AMI correlated with the extent of irreversible myocardial injury determined by LGE at 6 months (*r*^2^ = 0.42, *P* = 0.001, *Figure 2D*) and weakly with the volume of acute ischaemia, as measured by oedema on T2-weighted magnetic resonance imaging (*r*^2^ = 0.29, *P* = 0.021; *Figure 2E*).

Having confirmed similarities in the elevation of monocyte subtypes acutely after myocardial infarction, we next sought to evaluate functional characteristics, as evidenced by alterations in patterns of gene expression.

### Gene expression profiling of peripheral circulating monocytes identifies differentially expressed genes in mice and humans

Unsupervised principal components analysis demonstrated a clear difference in groups in mice (Supplementary material online, *Figure S1A*). A similar effect was present in human subjects, though, as expected, due to the variability in infarct size, ischaemic territory, ischaemia time, and genetic heterogeneity, the group effect was less pronounced (Supplementary material online, *Figure S1B*).

**Figure 3:**
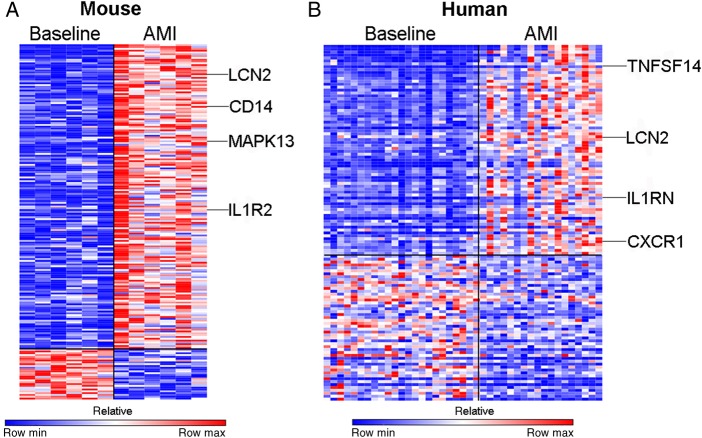
Acute myocardial infarction results in differential expression of genes in circulating monocytes in both humans and mice. (*A*) Analysis of the transciptome in mouse monocytes revealed differential expression of 196 genes (fold change >2; *P* < 0.01) of which 168 genes were significantly up-regulated and 28 genes were significantly down-regulated (*n* = 6/group). (*B*) In humans, 122 genes were differentially expressed (fold change > 2; *P* < 0.01) of which 72 genes were significantly up-regulated and 50 genes were significantly down-regulated (*n* = 24, control group, *n* = 30, AMI group).

Supervised analysis of the transcriptome at baseline in comparison with 48 h following AMI in mouse monocytes revealed differential expression of 196 genes (fold change >2; *P* < 0.01) of which 168 genes were significantly up-regulated and 28 genes were significantly down-regulated (*Figure 3A*). A similar comparison in humans identified that 122 genes were differentially expressed (fold change >2; *P* < 0.01), of which 72 genes were significantly up-regulated and 50 genes were significantly down-regulated (*Figure 3B*). The change in gene expression was confirmed by PCR in selected genes in both mice and human studies (Supplementary material online, *Figures S2* and *S3*).

### Monocyte transcriptional response in acute myocardial infarction is conserved in mice and humans

**Figure 4:**
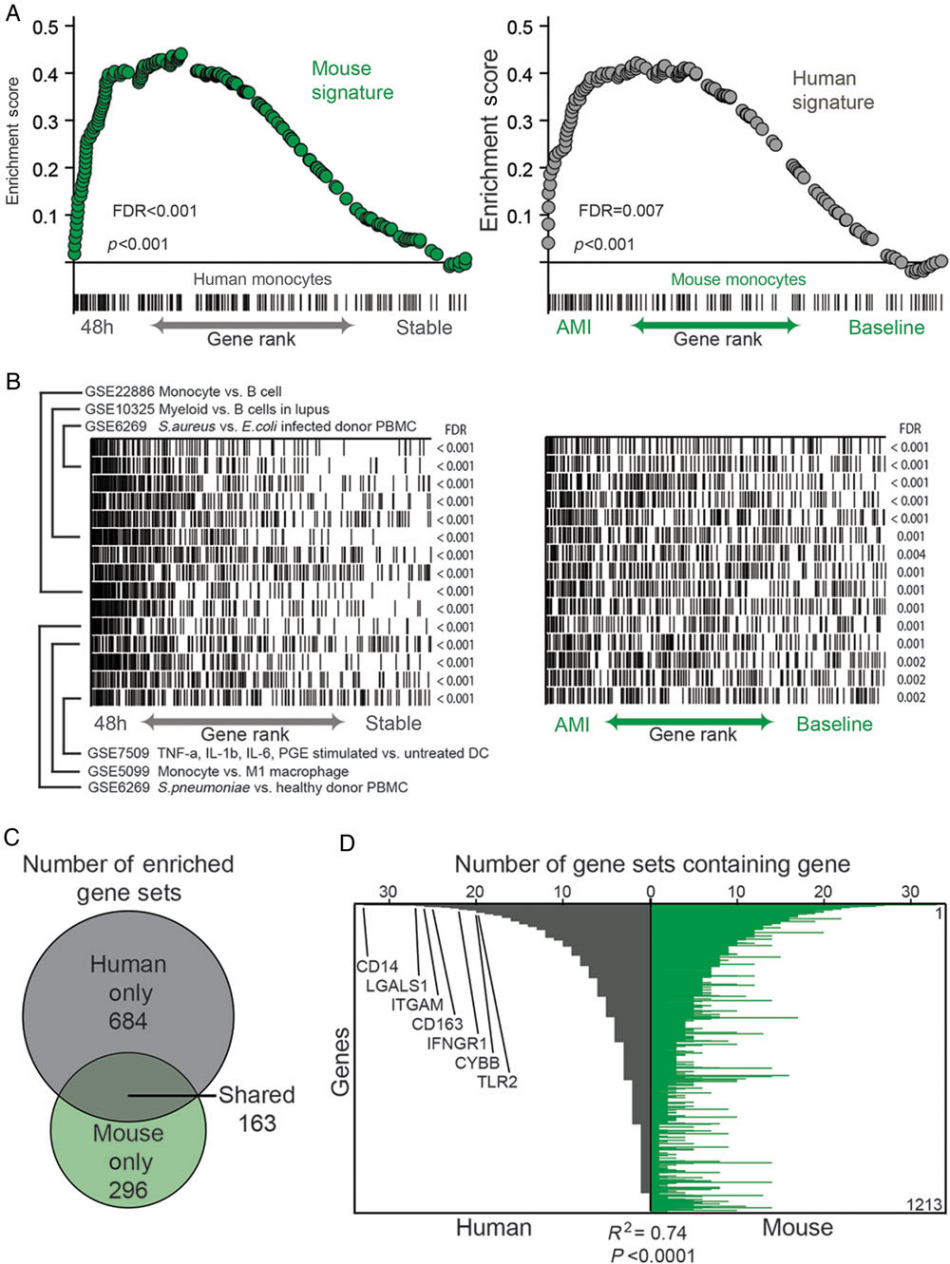
Monocytes from myocardial infarction in mouse and human are transcriptionally similar. (*A*) Mouse monocyte gene signature in acute myocardial infarction strongly enriches in the ranked gene list of human blood monocytes 48 h following infarct compared with the stable state (left) and *vice versa* (right). (*B*) Overlap of genes in same top enriching gene sets with the ranked gene list of the infarct vs. baseline state of monocytes in human (left) and mouse (right). (*C*) Number of gene sets in ImmuneSigDB v1.0 strongly enriching (FDR < 0.01) in infarct human and/or mouse monocytes. (*D*) Representation of genes in the leading edges of the 163 shared enriched gene sets. Genes were ranked from most to least represented in human dataset and shown is the number of times that gene appears in the leading edge of the enriched gene sets. Similarity of genes enriching in leading edges of enriched gene sets in both organisms was assessed by Spearman correlation.

Having identified individual genes that were significantly differentially expressed following AMI in peripheral circulating monocytes, we then investigated whether these observed changes conformed to similar patterns in mice and humans. In order to do this, we generated two ranked gene ‘signatures' (*n* = 200 genes) of the most up-regulated genes in circulating monocytes following AMI, one in the mouse model and one in humans. Gene set enrichment analysis revealed significant enrichment of the mouse signature in the ranked list of genes up-regulated in human monocytes after AMI (*P* < 0.001, FDR < 0.001, *Figure 4A*). Similarly, a signature of genes differentially expressed in humans revealed significant enrichment in the ranked list of genes up-regulated in mouse monocytes after AMI (*P* < 0.001, FDR < 0.01, *Figure 4A*). This comparison of the primary data sets shows that the pattern of gene expression induced in monocytes following myocardial infarction is significantly conserved between species.

We then identified the biological processes present in human and mouse monocytes following AMI by testing each monocyte expressed dataset for up-regulation of a curated compendium of ∼2000 immune-related signatures.^28^ We identified 163 gene sets that were enriched in both species following AMI, including gene sets related to myeloid lineage, bacterial infection, and cytokine stimulation of innate cells (*Figure 4B* and *C*).

We next examined the representation of genes that lay in the leading edges of gene sets showing enrichment in both species. The leading-edge genes of an enriched gene set are those that contributed most significantly to the enrichment score, and reflect the major ‘drivers’ of enrichment. The leading-edge genes showed striking similarity between the two species (*Figure 4D*), again suggesting that the transcriptional profile of monocytes following AMI in human and mouse shared common biological characteristics. Several of the most commonly represented genes in both human and mouse included *CD14*, *LGALS1*, *ITGAM*, *CD163*, *IFNGR1*, *CYBB*, and *TLR2* with roles in monocyte inflammation, cell-to-cell signalling, and cellular proliferation (*Figure 4D*).

### Gene set enrichment analysis identifies functional characteristics of circulating monocytes in acute myocardial infarction

**Figure 5:**
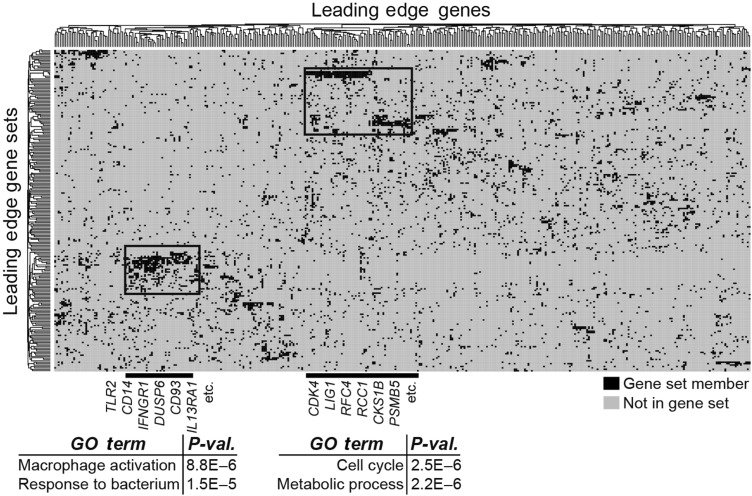
Leading-edge analysis identifies inflammation and cell cycling as novel biological states underlying the monocyte response to acute myocardial infarction. Leading-edge analysis of most significantly enriching gene sets (FDR < 0.01) in mouse monocytes following myocardial infarction reveals metagenes involved in inflammatory and cell cycle processes. Representative genes in these metagenes are listed in Supplementary material online, *Table*.

To gain further insights into the biological function revealed by these enrichments, we performed leading-edge analysis of the most significantly enriched gene sets (FDR < 0.01) corresponding to the most differentially expressed genes in mouse monocytes, following myocardial infarction. We found two clusters of *genes* (vertical clustering) that were represented in multiple immune *gene sets* (horizontal clustering) following AMI. This suggests that these genes form part of transcriptional modules of coordinately regulated genes that are up-regulated in mouse monocytes. We functionally annotated these clusters of up-regulated genes using Gene Ontology (*Figure 5*), and found significant over-representation of genes related to inflammation (including *TLR2*, integrins, and chemokines including *CCR1*; Supplementary material online, *Tables S3* and *S4*) and cell cycle (including cyclins, annexins, and tetraspanins; Supplementary material online, *Tables S5* and *S6*).

### Validation of transcriptional findings

**Figure 6:**
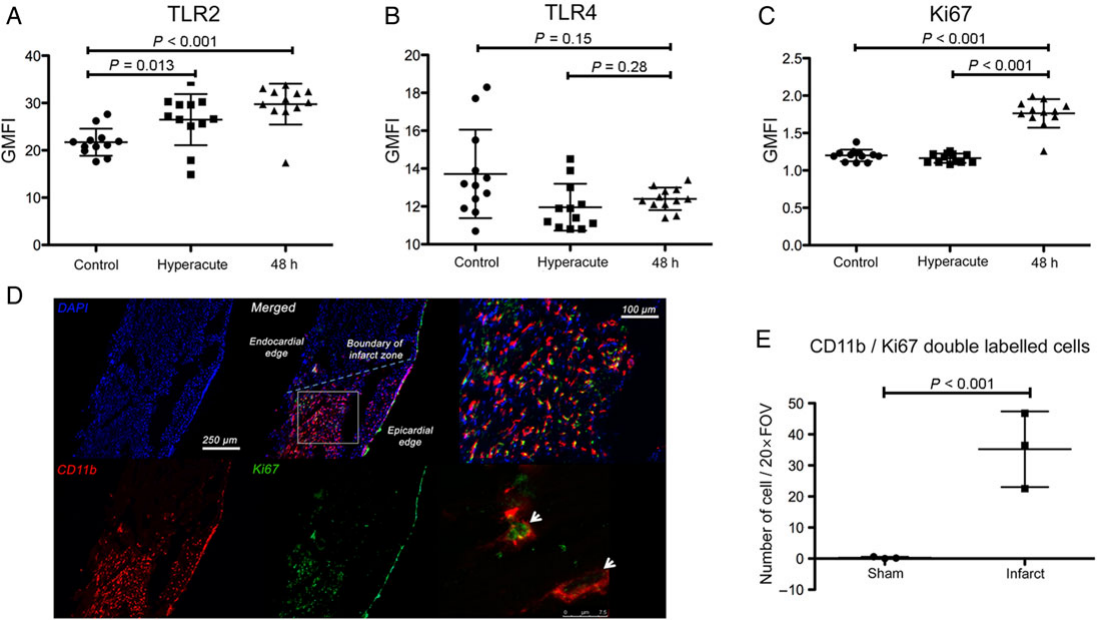
Inflammatory and mitosis pathways are up-regulated in circulating monocytes *en route* to inflamed myocardium. (*A*) Flow cytometry of peripheral circulating monocytes 48 h following acute myocardial infarction in humans identified a significant increase in the expression of TLR2 (*P* < 0.001) but not TLR4 (*P* = 0.14, *B*) (*n* = 12/group). (*C*) Ki67 expression was significantly up-regulated in monocytes at 48 h following injury but not hyperacutely (at presentation) in comparison with controls (*P* < 0.001) (*n* = 12/group). (*D* and *E*) Immunohistochemistry of mouse hearts (blue: DAPI, red: CD11b, green Ki67) following acute myocardial infarction confirmed a significant increase in the number of leukocytes expressing Ki67 (white arrows) (*P* < 0.001) indicating up-regulation of mitosis pathways in infarcted myocardium (*n* = 3/group). Data are represented by mean ± standard deviation.

Up-regulation of proliferation-associated genes was a prominent feature of the transcriptional profile of monocytes following AMI in both humans and mice. To confirm this finding, we undertook fluorescence-activate cell sorting (FACS) analysis of peripheral blood mononuclear cells stored from the same patients at the 48 h time point for the proliferation markers (Ki67 and cyclin) and markers of inflammation (TLR-2 and TLR-4). As predicted from the leading-edge analysis, there was (i) a significant increase in TLR-2 at protein level (*P* < 0.001, *Figure 6A*), and (ii) no change in TLR4 (*P* = 0.14, *Figure 6B*). Ki67, a marker of cell proliferation, was increased by 70% (*P* < 0.001, *Figure 6C*) in monocytes 48 h after MI. Given the elevation in Ki67 in peripheral monocytes, we next analysed mouse hearts to establish whether monocyte-derived cells in the myocardium *at this very early time point* already showed evidence of proliferation. In sham-treated mice, there were small numbers of CD11b^+^ cells of which virtually none were Ki67^+^. Following AMI in mice, there was a massive (130-fold), increase in the number of CD11b^+^ cells within the myocardium, with abundant Ki67-expressing CD11b^+^ leukocytes (*P* < 0.001 vs. sham-operated hearts, *Figure 6D* and *E*).

## Discussion

Data from mouse models strongly implicate the innate immune system, and in particular monocytes as critical to initial inflammatory and latter reparative processes following AMI.^2,4–7^ There is an early influx of Ly6c^hi^ monocytes, which are inflammatory,^15^ with a later preponderance of Ly6c^low^, F4/80^high^ macrophages, which contribute to myocardial repair.^3^ The relatively sparse data from human studies in the context of AMI are consistent with these findings,^14^ but analyses have been confined to a small number of cell surface markers that provide limited insight into function and are not biologically useful.^9,10^

Whilst previous studies have investigated the monocyte transcriptome, these have been limited to cells in the resting state.^15,34^ Here we present analyses of the changes in the blood monocyte transcriptome in mice and humans early after AMI. Through analysis of the changes in patterns of gene expression, we have been able to gain further functional insights into monocyte biology and this work contributes two potentially important findings. Firstly, the transcriptional response following AMI in monocytes between mice and humans appears to be largely conserved. This provides a validation of mouse models to study innate immune responses following acute MI. This is important since conservation across species cannot be assumed. For instance, recent comparisons of changes in gene expression in models of sepsis showed differences in gene expression between humans and mouse models. Secondly, current thinking suggests that monocytes act in ‘response mode’ whereby monocytes enter the circulation and ‘patrol the vasculature…. before being recruited to sites of inflammation*.*’.^35^ Although the absolute number and proportion of Ly6c^hi^ monocytes in circulating blood is well known to increase in AMI, changes in their function, implying a form of ‘programming’ prior to arrival at the site of inflammation have not previously been demonstrated. Here we show that, in mice and human monocytes, patterns of gene expression associated with inflammation and proliferation are switched on *prior* to their infiltration of injured tissue.

Whilst monocytes might be programmed in the blood, the recent observation that a cardiosplenic axis exists in humans^14^ (supporting findings in experimental models^15^) suggests that these cells could also be programmed in a different pool and ‘released’ in response to injury. Of relevance to this concept, observations from platelet studies have demonstrated that the expression of MRP-14 was found to be significantly up-regulated in circulating platelets of patients prior to myocardial infarction.^36^ Our work therefore suggests that a ‘pre-programmed state’ may not just be limited to platelets but may also extend to monocytes.

Whilst our data do not exclude the possibility that naïve monocytes are recruited and then activated in the myocardium and reintroduced into the blood, this is not likely given what is already known of (i) the timing and (ii) large net flux of monocytes from spleen to heart in AMI. Furthermore, recent studies in experimental models show that once in the infarcted myocardium, Ly6c^hi^ monocytes differentiate *in situ* into Ly6c^hi^, F4/80^+^ macrophages.^3,37^

In keeping with previous reports, we show that AMI leads to a small but statistically significant increase in the number of Ly6c^high^ monocytes. However, it is notable that in both the resting state and after AMI, Ly6c^high^ monocytes predominate. Consequently, analysis of Ly6c^high^ monocyte numbers provides virtually no functional insight. We therefore undertook whole transcriptome analyses, using a non-selective, unbiased approach that was not constrained by examination of one or other monocyte subsets. Better to understand changes in gene expression, we employed GSEA. Gene set enrichment analysis features a number of advantages when compared with single-gene methods. Firstly, it provides a structure for interpretation by identifying pathways and processes. Rather than focusing on highly regulated single genes (which can be difficult to interpret mechanistically), GSEA focuses on gene sets, which tend to be more reproducible and more interpretable. Secondly, when the members of a gene set exhibit strong cross-correlation, GSEA boosts signal-to-noise ratio making it possible to appreciate the contributions of even modest changes in individual genes. Thirdly, ‘leading-edge analysis' can help define gene subsets to elucidate key players and identify critical biological processes.^28^

In the first instance, we show conservation between the mouse and human monocyte response to AMI. This is important since it provides surety for mechanistic studies in mice and for the development of new therapies. There were, however, also differences between species, with some biological processes (e.g. complement activation) up-regulated in the mouse but not in humans. Identification of inter-species differences is also important since they may anticipate discrepant outcomes associated with targeting particular process, including the complement cascade in clinical studies.^38^ Conversely, we also identified pathways, e.g. integrin-linked kinase pathway that has roles in cell migration, adhesion, and signalling,^39^ which have not been described previously in this pathology possibly due to a lack of function in this setting in mice.

Leading-edge analysis identified single genes *CD14*, *LGALS1*, *ITGAM*, *CD163*, *IFNGR1*, *CYBB*, and *TLR2* with roles in inflammation and cellular proliferation as central biological processes to the monocyte response to AMI in humans. Cellular proliferation within target tissue has recently, and unexpectedly, been shown to be critical to monocyte mediated reparative processes following AMI in experimental models^3^ and in atherosclerotic plaque progression^40^ and thus might represent drug-sensitive targets.

Gene expression profiling methods have provided a clearer view of the level of molecular heterogeneity that underlies pathologies including cancer,^41^ cardiomyopathy,^42^ cardiac transplantation,^43^ vaccine development^44^ and in the diagnosis of giant cell myocarditis.^45^ These technologies have also been used in the identification of novel targets for drug development^46^ and in predicting response to treatment.^47^ By applying these methodologies to the setting of AMI, we have identified a number of biological pathways that are up-regulated in monocytes following AMI in both mice and humans. The specific targeting of immune cells in AMI is an attractive therapeutic option; however, clinical trials that have adopted a broad strategy of inhibiting inflammation have been disappointing to date.^48,49^ Targeted therapies based on patient stratification on the basis of target cell transcriptome is appealing, identifying patients who have most to gain from specific biological therapies and those that would only be subjected to undue risk.

### Limitations

Although the gene sets were obtained from large compendia of published data, they are inevitably incomplete in terms of the totality of possible biological states and also biased by the focus of experimental data submitted. Therefore, the interpretations of the transcriptome should be judged in that context and not considered definitive. It is also unlikely that the monocyte transcriptome is static, changes will occur over time, perhaps in response to new signals, and thus the peripheral monocyte transcriptome may not necessarily reflect the transcriptome following infiltration into ischaemic myocardium.

This investigation was undertaken at a single time point, in part informed by the data for mice in the literature, although observed changes may vary between species at different timepoints. Subsequent studies will extend current evaluations to later timepoints.

The magnitude of the monocyte response correlated with extent of LGE at 6 months but only weakly with early myocardial oedema. This may reflect a monocyte response that is driven by the extent of tissue that has undergone irreversible injury. However, a recent study has shown a biphasic accumulation of myocardial oedema that diminishes the utility of a single measurement to indicate extent of acute ischemic injury.^50^

## Conclusions

This study demonstrates that the peripheral blood monocyte response following AMI is conserved between species both phenotypically but also at the level of the transcriptome. We show that circulating monocytes are ‘programmed’ with a number of biological processes up-regulated prior to their arrival at sites of myocardial injury and have identified mitosis a particularly important, but little recognized contributor to early inflammation and possibly latter reparative processes. Although monocyte proliferation has recently been shown to be important several days after AMI in mice,^3^ our data suggest both pre-activation and early proliferation at the site of infarction.

## Supplementary material

Supplementary material is available at *European Heart Journal* online.

## Funding

This work is supported by the Wellcome Trust, the British Heart Foundation, the National Institutes of Health (NIH), and the National Institute for Health Research (NIHR) Oxford Biomedical Research Centre. There are no relationships with industry. Funding to pay the Open Access publication charges for this article was provided by The Wellcome Trust.

**Conflict of interest:** none declared.
